# Bibliography

**Published:** 1905-07-15

**Authors:** 


### BIBLIOGRAPHY.

Messrs. Lea Bros. & Co. announce an entirely new edition of Gray's anatomy. Probably no text book on this subject has ever approached in popularity this book, it has been the standard for fifty years, although many attempts have been made to supplant it. The fact that Dr. DaCosta of Philadelphia, has spent two years in completely revising it, and the publishers have spared no expense in order that the work may take on new life, is sufficient evidence that Gray's anatomy will retain its place for many years to come. New type, new matter, new illustrations and a real revision ought to produce a valuable work.

---

Notes on Dental Porcelain, by Dr. D. Walter Gilbert, is a new publication from the S. S. White Dental Manufacturing Company. It is a compact little book of 125 pages, well printed and illustrated. The author has endeavored to set forth the methods of manipulating the various grades of porcelain used in dentistry. Of course, in so small a work it could not be expected that the whole subject could be adequately discussed. It is surprising, however, to note how much of the principle and details the book contains. It is really the best thing of the kind on the book market, and should be in the library of every porcelain worker.

The comprehension of the contents of this little work will start anyone in the right way to manipulate successfully dental porcelains.

